# “InMotion”—Mixed physical exercise program with creative movement as an intervention for adults with schizophrenia: study protocol for a randomized controlled trial

**DOI:** 10.3389/fnhum.2023.1192729

**Published:** 2023-07-05

**Authors:** Hanna Poikonen, Anna Duberg, Mats Eriksson, Martin Eriksson-Crommert, Majja Lund, Margareta Möller, Mussie Msghina

**Affiliations:** ^1^Professorship for Learning Sciences and Higher Education, Department of Humanities, Social and Political Sciences, Swiss Federal Institute of Technology Zurich (ETH Zürich), Zürich, Switzerland; ^2^University Health Care Research Center, Faculty of Medicine and Health, Örebro University, Örebro, Sweden; ^3^Faculty of Medicine and Health, School of Health Sciences, Örebro University, Örebro, Sweden; ^4^Faculty of Medicine and Health, School of Medical Sciences, Örebro University, Örebro, Sweden; ^5^Department of Psychiatry, School of Medical Sciences, Faculty of Medicine and Health, Örebro University, Örebro, Sweden; ^6^Department of Clinical Neuroscience, Karolinska Institute, Stockholm, Sweden

**Keywords:** schizophrenia, physical activity, brain imaging, Motion Capture, physical therapy, rehabilitation medicine, dance

## Abstract

**Background:**

Schizophrenia is among the world’s top 10 causes of long-term disability with symptoms that lead to major problems in social and occupational functioning, and in self-care. Therefore, it is important to investigate the efficacy of complementary treatment options for conventionally used antipsychotic medication, such as physical training, and psychosocial interventions.

**Objective:**

To combine aerobic and strength training with cognitive, emotional and social stimulation in one intervention for people with schizophrenia and test the feasibility and effects of this intervention.

**Methods:**

The study is a mixed-method randomized controlled trial to evaluate the effects of a 12-week intervention for adults with schizophrenia. The treatment group (30 participants) will receive the intervention in addition to standard care and the control group (30 participants) only standard care. The intervention consists of 24 biweekly sessions with a duration of 60 min. The pre-test (weeks from 4 to 2 prior to the intervention) and post-test (week 12) include clinical measure (PANSS), quality of life, social performance, movement quantity, brain function and eye tracking measures. In addition, a treatment subgroup of 12–15 participants and their family member or other next of kin will complete a qualitative interview as a part of their post-test. Two follow-up tests, including clinical, quality of life, brain function and eye tracking will be made at 6 and 12 months from the completion of the intervention to both study groups. The primary outcome is change in negative symptoms. Secondary outcome measures include general and positive symptoms, quality of life, social performance, movement quantity, brain function and eye tracking. Explorative outcome includes patient and family member or other next of kin interview.

**Results:**

Pilot data was collected by June 2023 and the main data collection will begin in September 2023. The final follow-up is anticipated to be completed by 2026.

**Conclusion:**

The InMotion study will provide new knowledge on the feasibility, efficacy, and experiences of a novel intervention for adults with schizophrenia. The hypothesis is that regular participation in the intervention will reduce clinical symptoms, normalize physiological measures such as brain activation, and contribute to new active habits for the participants.

**Trial registration:**

ClinicalTrials.gov, identifier NCT05673941.

## 1. Introduction

Schizophrenia is among the world’s top ten causes of long-term disability (GBD 2016 Disease and Injury Incidence and Prevalence Collaborators, 2017; Global Burden of Disease Collaborative Network, 2020). The major symptoms of schizophrenia include hallucinations, delusions, affective flattening, apathy and cognitive impairment. These symptoms lead to major problems in social and occupational functioning, and in self-care. Roughly 0.5–1% of the population is affected by schizophrenia, with similar rates across different continents, cultural groups, and sexes. The etiology of schizophrenia is not fully known but malfunctioning of the dopaminergic system is one of the leading hypotheses. Currently antipsychotic medications that modulate the dopaminergic system are exclusively the only available pharmacological treatment options for schizophrenia (Xu and Yang, 2022). However, treatment with antipsychotic medications is far from optimal in improving the symptoms of schizophrenia and can cause substantial adverse events that negatively impact patients’ physical health and quality of life (Stroup and Gray, 2018). While psychopharmaceuticals are effective in improving positive symptoms, their effectiveness on negative symptoms is limited. Therefore, it is important to investigate the efficacy of complementary treatment options, such as physical training and other psychosocial interventions, to reduce the impact of negative symptoms and enhance quality of life. Previous studies show promising effect of dance movement therapy or physical exercise on negative symptoms and quality of life (Röhricht et al., 2011; Lee et al., 2015; Dauwan et al., 2016; Martin et al., 2016; Gökcen et al., 2020). However, the transfer effects of such bodily interventions to cognition or emotion regulation, and their underlying brain processes, are studied to a lesser extent.

### 1.1. Physical training in people with schizophrenia

People with schizophrenia have a substantially lowered life expectancy, reduced cardiorespiratory and muscular fitness and increased risk of cardiovascular disease and metabolic syndrome in comparison to the general population (Vancampfort et al., 2015a,2016a,2017a; Sugai et al., 2016; Correll et al., 2017). They also have lower physical activity and higher level of sedentary lifestyle than the general population (Lahti et al., 2012; Crump et al., 2013; Vancampfort et al., 2017b). Eight systematic reviews have reported benefits of exercise for people with schizophrenia. The benefits cover general health outcomes, specific psychiatric symptoms and comorbid disorders (Soundy et al., 2014; Firth et al., 2015; Martin et al., 2017; Keller-Varady et al., 2018), cognitive functioning such as working memory, planning and problem solving (Sanada et al., 2016; Firth et al., 2017a,b) and cardiorespiratory fitness (Vancampfort et al., 2015b). However, some of these studies have been criticized for small sample sizes and poor reporting of the intervention characteristics. Overall, the systematic reviews highlighted that group exercises result in substantially better outcomes than solitary training (Firth et al., 2015, 2017a) and reduce the dropout rate (Firth et al., 2015; Vancampfort et al., 2016b). In addition, further improvements in outcomes and dropout rate could be seen when the interventions were facilitated by experienced professionals (Firth et al., 2015).

Mixed exercise program that combines aerobic, flexibility and resistance training is suggested to be effective in decreasing the symptoms of schizophrenia and increasing cardiorespiratory fitness and muscular strength (Martin et al., 2017; Girdler et al., 2019). However, studies suggest that better physical fitness is not associated with improved cognitive performance over 2 years, nor did the benefits on muscular endurance extend into a 3-month follow-up examination period (Cheng et al., 2017). There are also contradictory results regarding whether physical exercise interventions enhance plasma brain-derived neurotrophic factor (BDNF) in people with schizophrenia (Ikai et al., 2014; Kim et al., 2014; Kimhy et al., 2015; Firth et al., 2017b), which is suggested to be the underlying factor for the beneficial changes in brain structure and functional connectivity gained through exercise interventions for people with schizophrenia. The most consistent evidence suggests performing at least 150 min of moderate to vigorous aerobic exercise per week may be optimal in decreasing the symptoms of schizophrenia (Vancampfort et al., 2012).

### 1.2. Body awareness, emotions, and social cognition in people with schizophrenia

Expressive therapies, through dance, music or drama, emphasize the human body as the primary means of communication and expression (Sherwood, 2008). Dance movement therapy has been used for people with schizophrenia since 1940s initiated by Marian Chace and Trudy Schoop (Bryl and Biondo, 2021). Several techniques of dance movement therapy, like mirroring, body action and group interaction, have been used in enhancing body awareness, agency for own body, attention, and emotion recognition and regulation of people with schizophrenia (Bryl and Biondo, 2021). Qualitative, quantitative and mixed methods studies suggest that dance movement therapy can improve positive and negative symptoms of schizophrenia (e.g., Lee et al., 2015; Martin et al., 2016; Bryl et al., 2020; Millman et al., 2021).

Mirroring movement is a core technique of the Chacian approach on dance movement therapy in which the therapist creates empathic movement reflections in an interaction with the patient to connect deeply and authentically (Bryl and Biondo, 2021). Chace believed that by recognizing and recreating a meaningful gesture, posture, or simple movement at the right moment, trust in the therapeutic relationship could be established, which further, could lead to the patient’s willingness and ability to communicate difficult or repressed feelings and thoughts (Klingberg and Buchkremer, 2000).

Many people with schizophrenia suffer from symptoms of disembodiment, body image disturbances and deficits in the feeling of being an agent in their own bodies and lives (Priebe and Röhricht, 2001; Maggini and Raballo, 2004). Body image refers to a mental representation of the body which is influenced by several factors, like cognitive and affective variables, cultural and social norms and psychopathology (Slade, 1994). Body awareness refers to the perceptual capacity and the distinction between the information obtained from the sensory organs, like the proprioceptors in the muscles and joints. Body awareness also involves awareness of one’s inner feelings and ability to be attentive to their inner feelings and moods (Givehki et al., 2018). Agency includes the sensation that one causes their own action and the consequences of those actions (Renes et al., 2013).

Working with and enhancing body awareness, movement and agency for own body and action are the core elements dance movement therapy (Bryl and Biondo, 2021). In dance movement therapy with people with schizophrenia, it is documented that bodily disorganization, which is one of the positive symptoms of schizophrenia observed as unsynchronized or contradictory movements in various parts of the body, often mirrors cognitive disorganization and depersonalization (Wade et al., 2017). Negative symptoms, which include affective flattening, diminished expressiveness, reduced mobility, rigidity of movement and asociality (Wade et al., 2017), have been successfully alleviated with dance movement therapy (Röhricht et al., 2011; Lee et al., 2015; Martin et al., 2016; Gökcen et al., 2020). Qualitative results on basic body awareness therapy with people with schizophrenia show that good balance and posture are often connected with increased self-esteem and feelings of security (Hedlund and Gyllensten, 2010). The same participants also experienced an increased sensory awareness and ability to be more mentally present. They also reported to be in better contact with the surroundings.

For people with schizophrenia, a qualitative study on dance movement therapy suggested that group exercises through dance may transfer to their daily life (Bryl et al., 2020). In general, the group setting is commonly used in dance movement therapy (Klingberg and Buchkremer, 2000; Jones et al., 2012; Bryl et al., 2020). Creative movement in a group setting is shown to reduce negative symptoms and enhance symptom management and social connection in people with schizophrenia (Bryl and Biondo, 2021).

Research on dance movement therapy for people with schizophrenia suggests enhanced attention and self-regulation (Davis, 1981; Martin et al., 2016) whereas studies on exercise for people with schizophrenia report contrasting results on the benefits of exercise to cognition (Dauwan et al., 2016; Sanada et al., 2016; Firth et al., 2017a,b). In addition, the Chacian approach of dance movement therapy suggests that associations between movements and corresponding emotions can be used for emotion recognition and regulation (Bryl and Biondo, 2021).

Taken together, recent results suggest that dance-movement therapy and body psychotherapy may be effective in reducing both negative and psychotic symptoms in individuals with schizophrenia, as well as improve psychosocial functioning and ability to control anger (e.g., Lee et al., 2015; Martin et al., 2016; Bryl et al., 2020; Millman et al., 2021). In addition, recent qualitative results suggest that dance-movement therapy enhance interpersonal relationship skills, sense of social integration, emotional support, and coping strategy in symptom management (Bryl et al., 2020).

### 1.3. Brain and behavior

Behavioral and brain imaging studies may help to explain the bodily disconnection people with schizophrenia display. A disturbed implicit sense of the bodily self in people with schizophrenia is traced partly to deficits in the motor representation in the brain related to their bodily self (Ferri et al., 2012). The brain functions of people with schizophrenia also seem to have altered processes beyond motor regions. Ebisch et al. (2014) showed in their fMRI study that people with schizophrenia have an aberrant functional interaction of premotor cortex and insula with cingulate cortex. These brain regions are known to be central in mediating self-experience referring to one’s own subjective experience as opposed to objective observation of someone else’s experience. On the other hand, people with first episode psychosis show a disproportionally strong coupling between the posterior and anterior parts of the insula (Ferri et al., 2012). The former is related to awareness of bodily sensations through connection with sensorimotor cortex, and the latter is associated with the integration of cognitive and emotional responses (Damasio et al., 2000; Critchley et al., 2004; Craig, 2009). Taken together, these results suggest an imbalance in the processing between internally and externally guided information and its abnormal integration with self-referential processing in patients with schizophrenia.

Several empirical studies suggest that disturbances in self-experience and self-monitoring could be closely associated with anomalies in the self-other relationship (Ihnen et al., 1998; Blakemore et al., 2000; Voss et al., 2010). Because cortical midline and premotor regions also have complementary functions in relating the self to its social environment (Gallese, 2003; Johnson et al., 2006; Uddin et al., 2007; Caggiano et al., 2009), altered interactions between these regions likely also disrupt some aspects of social cognition. Indeed, people with schizophrenia with high self-monitoring skills were reported to have better social skills than the ones who showed lower self-monitoring skills (Ihnen et al., 1998).

Low tolerance for stress and stimuli is a common symptom within people with schizophrenia (Mc Gorry, 2005; Sestito et al., 2015). Results showing a similarity in facial muscle activation for smile and cry stimuli suggest that people with schizophrenia may have a disruption of the automatic, low-level processes related to facial mimicry (Sestito et al., 2013). Further, this disruption may foster their sense of detachment and increase disconnection between the “expressed” and the “felt” self (Aghevli et al., 2003; Sestito et al., 2013). This growing disconnection crucially creates a sense of distance—and even alienation—from their own experience, ending up in a scission and fragmentation within the self. One way how embodied interventions which include body awareness and/or emotional expression, such as dance-movement therapy or basic body awareness therapy, may improve the clinical symptoms of people with schizophrenia is by re-generating the connection to one’s own body and emotions.

### 1.4. Possible benefits of mixed physical exercise with creative movement in schizophrenia

Conventional occupational or physical therapy in people with schizophrenia does not include emotional expression. On the other hand, dance-movement therapy and body psychotherapy focus on emotional, social, cognitive, and physical integration of the individual. They do not primarily aim for the physical benefits that aerobic training, conducted at a moderate to intense level around 60% and higher of the maximum heartrate, is known to have. Nor do they aim primarily for gaining muscle strength, the training of which utilizes body weight with repetitions and progression in load and complexity over time.

There is a growing interest in the mechanics through which motor-related brain processes influence in emotional and cognitive processes. Several studies show how creative movement, such as dance, shapes the brain structure and function in both the general population and trained dancers (Karpati et al., 2015) and modifies the functions in the motor-related brain regions (Calvo-Merino et al., 2005; Cross et al., 2006, 2009; Pilgramm et al., 2010). These changes may occur through the dopaminergic and serotonergic systems, the function of which is shown to balance in physical and rewarding (joyful) activity, paired with the reduction of the stress hormones such as cortisol. Continuous movement and shared joy are characteristic for the creative physical exercise which might help to engage people with schizophrenia who tend to have low motivation for physical activity (Bryl et al., 2020; Bryl and Biondo, 2021).

Positive influence of regular dance training is shown to go beyond the improvement of physical abilities to the alleviation or prevention of symptoms in clinical populations. Different dance trainings in people with stroke, Parkinson or dementia are shown to increase physical abilities such as balance, muscle strength and smoothness of movement sometimes even more efficiently than conventional physiotherapy. Such dance trainings are also shown to improve quality of life, mood, emotional expression, and other behavioral measures (Hackney et al., 2012; [Bibr B3][Bibr B81]). In mixed exercise program with creative movement, bodily awareness, action and reaction are fundamental factors when adapting to the environment, music and the movement of the fellow-movers (Prabhjot et al., 2015; McKay et al., 2016). In addition to physical health, engaging with joyful creative physical exercise may improve the brain processes involved in cognition, emotion, self-awareness, social interaction, and behavioral adaptation.

### 1.5. Aims and expected results

The primary aim of the study is to investigate the effects of mixed exercise program with creative movement and cognitive, emotional and social stimulation as add-on to ongoing pharmacological treatment, in treating negative symptoms of schizophrenia. Previous studies with dance movement therapy, body psychotherapy or physical exercise show reduction in negative symptoms of schizophrenia (Röhricht et al., 2011; Lee et al., 2015; Martin et al., 2016; Gökcen et al., 2020), and that the change in these symptoms can be measured with PANSS (Röhricht et al., 2011; Lee et al., 2015; Sabe et al., 2020; but see but see also Bryl et al., 2020 for qualitative but not significant quantitative results). Thus, our hypothesis is that the intervention will improve negative symptoms detectable with PANSS in comparison to the control group. The reduction is anticipated to correlate with changes in brain function and physical measures. In line with previous studies (e.g., Röhricht et al., 2011; Lee et al., 2015; Curcic et al., 2017; Sabe et al., 2020), we will measure both negative and positive symptoms as well as general psychopathology, i.e., total PANSS score and look separately at the three symptom classes.

The secondary aim is to evaluate potential change in (a) general and positive symptoms of schizophrenia, (b) quality of life, depression and social performance (c) levels of physical activity, physical function and general health indicators, and cortical aspects of (d) error detection and conflict resolution on the prefrontal cortex, (e) emotional induction and regulation on specific emotions on the frontal cortex, (f) full-body movement quantity and rigidity, (g) cognitive function such as memory and fluency of speak, and (h) to explore the participants experiences of taking part in the intervention and any experienced change from it. Similarly, experiences from their related parties (a family member or other next of kin, nursing staff) will be collected regarding the influence of the intervention in the participants (Bryl et al., 2020). Our hypothesis is that, in comparison to the control group, the intervention group will show (i) strengthened physical health, function and reduced sedentary behavior, (ii) improvement in cognitive control and emotion regulation (Davis, 1981; Martin et al., 2016) and changes in their neural correlates (Balconi et al., 2015; Popov et al., 2019), and (iii) increased mobility and reduced rigidity in movement in the schizophrenia patients over the intervention (Bryl and Biondo, 2021) which can be also seen as increased fluidity and quantity of movement measured with Motion Capture (Poikonen et al., 2018).

## 2. Materials and methods

### 2.1. Approval and registration

The study was approved by the Swedish Ethical Review Board in Uppsala, Sweden (No. 2022-03980) and is registered on ClinicalTrials.gov (No. NCT05673941). Any protocol modifications will be communicated to relevant parties.

### 2.2. Study design

This study, called InMotion, will be conducted in two phases: pilot study and main study.

In the pilot study, which finished in June 2023, two people with schizophrenia participated in the intervention of mixed exercise program with creative movement (InMotion intervention) with 2–3 instructors. Participants did not need so many instructors, but the pilot study was a good chance also for the instructors to practice guiding the intervention. Hence, three instructors participated in some of the pilot sessions. A family member or other next of kin could attend the movement sessions if the patient wished so. The intervention lasted 12 weeks including 60-min sessions twice a week, similar to the following main study. The following pre- and post-intervention data was collected: PANSS, CGI-S, CGI-I, PSP, CDSS, BAQ, EQ5D, blood pressure, resting heartrate, breathing frequency, waistline, BMI, postural stability, leg strength and accelerometer/everyday activity. Cognitive and brain imaging data were not collected at this phase due to logistical issues with the EEG-fNIRS equipment. However, the brain imaging measurement will be piloted separately with a person with schizophrenia.

**FIGURE 1:**
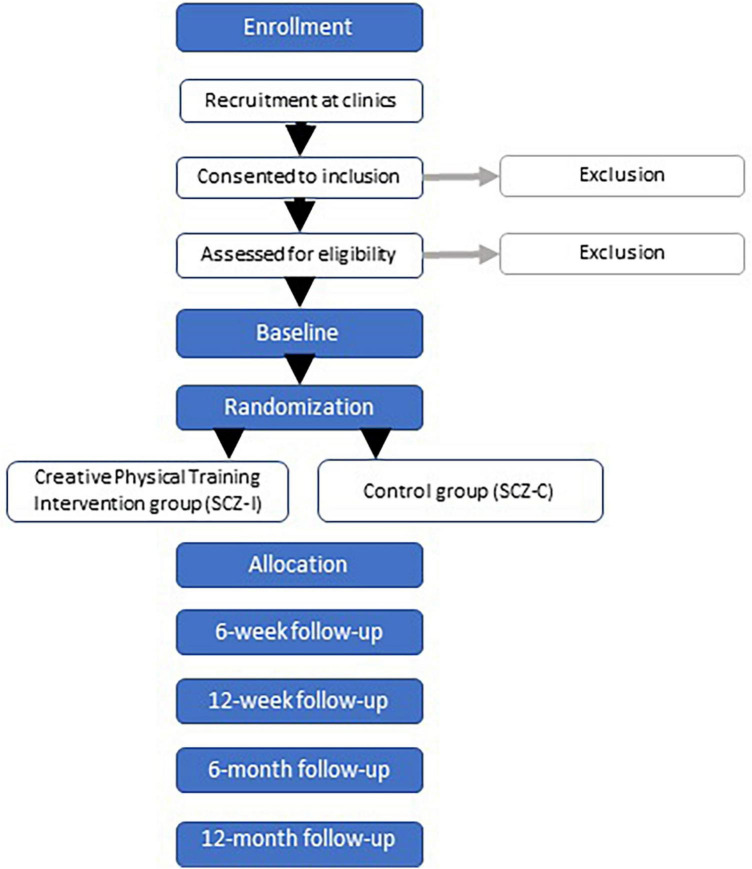
Flowchart of the protocol.

The main study will be a prospective randomized controlled trial (RCT) with 2 parallel groups: an intervention group (30 participants) and a control group (30 participants) of adults with schizophrenia or disorders with symptoms similar to schizophrenia, as described in section “2.3.1. Inclusion criteria.” The intervention consists of sessions on mixed exercise program with creative movement two times a week for 12 weeks in addition to standard medical care as described in section “2.9 Standard care.” Each group will include 4–6 participants under the guidance of two instructors. The control group receives standard medical care and gets access to the video-links after the 12-month follow-up. Most outcomes are measured at baseline, after 6 and 12 weeks, 6 and 12 months (see Figure 1 for details). At all follow-ups, data is collected for both the intervention and control groups. The timeframe of the main study is from autumn 2023 to spring 2025. The study adheres to the standard methodology of intervention research. This protocol is conducted according to the SPIRIT (Standard Protocol Items: Recommendations for Interventional Trials) guidelines (Chan et al., 2013). The study will be reported according to the CONSORT (Consolidated Standards of Reporting Trials) guidelines (Schulz et al., 2010), and COREQ (Consolidated Criteria for Reporting Qualitative Research; Tong et al., 2007).

### 2.3. Study population

According to national guidelines (Socialstyrelsen, 2018), schizophrenia includes positive and negative symptoms. The criteria provided in the current guidelines (Socialstyrelsen, 2018) and described in section “2.3.1. Inclusion criteria” is applied during the study inclusion at the beginning of the study. The study population (*N* = 60) includes adults diagnosed with schizophrenia or schizophrenia-like disorders according to the criteria presented below.

#### 2.3.1. Inclusion criteria

People aged 18–65 years with schizophrenia diagnosis or schizophrenia-like disorders, performing moderate to vigorous physical activity less than 150 min/week based on self-report, are included in the study. Outpatients with schizophrenia (F20), schizoaffective disorder (F25), and acute schizophrenia-like psychotic disorder (F23.2) with at least mild symptom severity (>21 on 7 negative items of PANSS) will be included.

#### 2.3.2. Exclusion criteria

People with current or recent history of substance abuse, bipolar disorder, severe autism, suicidal, severe physical illness, inability to read and understand the Swedish language, inability to participate in a group setting or patients who reported more than 150 min of physical activity at moderate to high level per week are excluded from the study. Patients with less than 21-point score on PANSS negative scale will be excluded.

### 2.4. Recruitment

Recruitment is planned to take place at an outpatient psychiatry center, where clinicians, such as psychiatrists, nurses, occupational therapists, social workers, physical therapists and assistants are asked to inform patients about the study and recruit them during their regular check-ups. Written and informed consent will be obtained from all participants. After written consent is received, the diagnosis according to the inclusion criteria will be verified in the medical records or by examination by a psychiatrist.

Schizophrenia patients and their legal guardians, who consent to the study and are eligible for the study according to the inclusion criteria, will be offered to proceed with the baseline measurement.

### 2.5. Modes of delivery

The intervention covers various themes through three different parts (4 weeks per part) with the aim to improve clinical symptoms, physical fitness, cognitive skills and emotion regulations. The intervention was developed by a team consisting of a neuroscientist, a psychiatrist, dance teachers and physiotherapists with previous experience in designing dance and movement interventions for different target groups with internalizing problems (Duberg et al., 2013), functional abdominal pain (Högström et al., 2022) and profound intellectual and multiple disabilities (Lundqvist et al., 2022). The intervention is further described in Table 1. The exercise is partly accompanied by instrumental music and recorded nature sounds and partly made in silence paying attention to one’s own bodily rhythm. The exercise consists of tasks related to cardio and strength training, balance, conscious breathing, body awareness, mental imagery (nature visualizations), flexibility, emotional expression, and movement anticipation together with the facilitator and fellow participants. All the tasks are practiced during a flow of 60-min exercise without any major breaks. To meet the needs of the participants in the group, adjustments and modifications will be presented when necessary. Complexity and physical intensity of the tasks will gradually increase over the 12 weeks intervention periods.

**TABLE 1:** Content of the biweekly 12-week intervention on mixed exercise program with creative movement for adults with schizophrenia.

Minutes	Section	Description	Purpose
		Gathering in a circle	Establishing a feeling of being included
2–4	Body scan	Scanning through the body parts Part 1 (moving), Part 2 (self-massaging and tapping), and Part 3 (moving)	Body awareness Hedlund and Gyllensten, 2010; Ferri et al., 2012; Martin et al., 2016; Gökcen et al., 2020
3–7	Gentle movement, flexibility (warm up/preparation)	Stretching arms, sides, neck, rotating shoulders, back, hip, and knees	Body awareness, increased blood-circulation and synovial fluid, stimulate mental presence. Hedlund and Gyllensten, 2010; Ferri et al., 2012; Martin et al., 2016; Bryl et al., 2020
8–10	Cardio	Reaching, jumping, walking, running	Strengthen cardio-vascular function. Soundy et al., 2014; Firth et al., 2015; Vancampfort et al., 2015b; Cheng et al., 2017
8–10	Choreography	Memorizing a dance sequence	Enjoyment, coordination, body awareness Firth et al., 2017a,b; Chen et al., 2018
7–9	Strength	Strength training for large muscle groups: legs, arms, back, core	Increase strength in large muscle groups. Martin et al., 2017
5–8	Balance	Walking carefully with one-leg-balances	Improve balance through increased proprioception and interoception. Hedlund and Gyllensten, 2010; Fuchs, 2018
4–6	Mirroring	Moving in synchrony with a partner/in a small group	Social interaction Hedlund and Gyllensten, 2010; Röhricht et al., 2011; Ebisch et al., 2014; Bryl et al., 2020; Gökcen et al., 2020; Bryl and Biondo, 2021
4	Emotional expression	Expressing with the face and body basic positive emotions: joy, tenderness, curiosity, gratefulness	Emotional expression Röhricht et al., 2011; Ebisch et al., 2014; Lee et al., 2015; Bryl and Biondo, 2021
4	Improvisation	Own improvisation with the guidance of the facilitator	Röhricht et al., 2011; Bryl et al., 2020
2–4	Body scan	Scanning the body and grounding back to the current moment	Body awareness Hedlund and Gyllensten, 2010; Ferri et al., 2012; Martin et al., 2016
Total duration: 60 min			

### 2.6. Randomization

The participants will be randomly divided into two groups, 30 patients who will receive the mixed exercise program with creative movement intervention in addition to continuing their conventional treatment plan and 30 patients who will only receive their usual treatment. Randomization in the main RCT study will be carried out in sealed envelopes after the baseline measurements. An external statistician will prepare the envelopes that will be delivered to the participants by the research team. Thus, the randomization will be blinded to the research team, meaning the research team will not know the group or identify of the participants when analyzing the data. Due to the nature of intervention studies, blinding toward the participants is not possible. Physical activity cannot be blinded, meaning that the patients know whether they are receiving the treatment or not.

### 2.7. Sample size

The sample size is calculated according to the primary outcome PANSS. For the primary outcome (improved negative symptoms according to PANSS after 12 weeks), the total number of required study participants was calculated (Curcic et al., 2017). Calculation was based on a minimum improvement of 15% on total PANSS score between the intervention and control group. With a power of 80% and a significance level of 0.05, we calculated the sample size to be *N* = 60 (30 intervention + 30 control participants), including a 25% dropout rate.

### 2.8. Intervention

The intervention is called “InMotion” to highlight physical activity as a natural part of positive lifestyle habits for adults with schizophrenia. Essential elements of the intervention will include a focus on physical exercise, enjoyment, socialization, and playful creativity in a safe environment. Physical activity is central in the intervention since previous result showed decreased BMI and increased condition (Martinsen and Taube, 2008; Cheng et al., 2017; Girdler et al., 2019), increased memory and social and problem-solving ability (Girdler et al., 2019). Body awareness training is combined with creative movement because of experiences from earlier studies on dance-movement therapy, movement therapy and dance, which have shown decreased schizophrenia-symptoms and increased ability to cope with remaining symptoms (Ren and Xia, 2013; Martin et al., 2016; Koch et al., 2019). When there are challenges with verbal language, social interventions that includes body, senses and action is particularly effective and studies have shown that structured, joyful movement led by instructor is important for people with schizophrenia (Martinsen and Taube, 2008; Kinou et al., 2013; Cheng et al., 2017; Millman et al., 2021). Moving in a group can strengthen the ability to understand one’s own body-language, as well as others. Emotional expression and creativity through movement can increase emotional richness and self-efficacy (Kinou et al., 2013; Millman et al., 2021).

The InMotion intervention shares some similarities with dance movement therapy. Our intervention has similar components especially with the approach by Chace (Bryl and Biondo, 2021). For example, mirroring movement is a core element of the Chacian approach on dance movement therapy (Bryl and Biondo, 2021). However, in our group intervention, mirroring is conducted by a fellow participant instead of the facilitator, whereas in dance movement therapy typically the therapist creates empathic movement reflections in an interaction with the patient (Bryl and Biondo, 2021). In our intervention, the fellow participants do not have a therapist’s education for mirroring a right gesture at the right time, but the mirroring interaction of two participants simulates real-life interactions with an emphasis on the embodied and non-verbal component of such interactions.

The group setting of our intervention is similar to the one used in dance movement therapy for people with schizophrenia (Klingberg and Buchkremer, 2000; Jones et al., 2012; Bryl et al., 2020). In addition, working with and enhancing body awareness, movement and agency for own body and action are some of the core elements of our intervention and dance movement therapy (Bryl and Biondo, 2021). What the InMotion intervention adds to the qualities of dance movement therapy, is the emphasis on physical cardio and strength training (Vancampfort et al., 2015b; Martin et al., 2017; Girdler et al., 2019). A specified amount of time is dedicated to increase the heartrate through fast walking, running, and dynamic movement which engage large muscle groups to achieve moderate to vigorous cardio-training. The intervention also includes strength-training using own body weight such as push-ups, squats and lunges.

### 2.9. Standard care

Standard care is based on the national guidelines includes regular outpatient visit at a minimum of once a year, treatment with an antipsychotic agent (>80% of those with standard care on one or the other antipsychotic agent), a once-a-year general check-up with blood samples including metabolic markers, among other things. This varies, however, according to individual patient need.

### 2.10. Instructors

The instructors are healthcare professionals such as counselors, physiotherapists or nurses with experience in physical interventions, and/or dance teachers (bachelor’s in dance pedagogy) with experience of meeting patients with mental health issues. Before starting the intervention in the main study, the instructors will attend a 1-day course regarding the target-group as well as the intervention administered by the research team. During this course, the instructors will receive practical instructions about the movement sessions according to the standardized program design and how to work with the target-group safely, supportively and ethically. The course will also cover shortly the theoretical background of schizophrenia and the mechanisms through which the intervention aims to improve the clinical, physiological and emotional condition of the target group. Written and digital informational material will be distributed by the research team. The material will be distributed not only with documents but also with informational videos made by the research team. The intervention coordinators will provide information regarding the practical components, choreography, and modifications as well as recorded movement exercises.

### 2.11. Procedures

When a patient at the psychiatry clinic is interested in participating in the main study, contact information will be transferred from responsible clinician to doctoral student, who will call the participant, give further information, answer questions if needed, and check inclusion/exclusion criteria. A meeting at the movement laboratory at the hospital will be set up with participant and psychiatrist who will do the PANSS, CGI-S, CDSS, and PSP as well as the doctoral student who will conduct physical tests and measures and test cognitive function combined with functional brain imaging and eye tracking. The baseline measures will take about 2.5 h with a break in between. Data on gender, age, years of education, way of living, years with schizophrenia diagnosis, medication, and smoking will be collected but no other sociodemographic information. The baseline measurements will be conducted 4 to 2 weeks prior to the beginning of the intervention. It may take a few weeks to form a group, and therefore, a 2-week window for the baseline data collection is needed. The participant will be instructed about the accelerometer, which is to be worn for 7 days to evaluate everyday activity. After baseline measures are finished, sealed envelopes will be given to the participants to randomize whether they will be part of the control or intervention group. The participants in the intervention group will be shown videos of the instructors and the training studio, to enable recognition and feeling of safety.

At session 2, 12, and 23 motion capture will be recorded. Those sessions will take place at the movement laboratory. Participants and one instructor will wear small reflex balls on six points: hands, elbows and knees, while doing the training session. Setting up will be done by an engineer at the movement laboratory or the doctoral student. On week 6 of the intervention the same procedure of tests, apart from the accelerometer, cognitive tests and functional brain imaging, will be repeated. After the last session, about two participants and their family members or other next of kin from each group will be purposefully selected for interview. The get a heterogenic group, we will strive for a variation regarding age, gender, participation rate and severity of disease. The participants and their family member or other next of kin will receive written and oral information about the qualitative study (including assurance of voluntariness and confidentiality), before signing a written consent. After finishing the intervention at week 12 all tests from baseline will be repeated in 1–2 weeks. This will also be done after 6 and 12 months, excluding the accelerometer.

The pilot study was conducted in the same way, except there was no control group or randomization procedure nor cognitive or brain imaging measures, nor will there be any follow-up tests in 6 or 12 months.

### 2.12. Locations

The intervention will most often take place in a training studio at the psychiatric clinic, an environment well known and safe to the participants. In detail, this applies to session 1, 3–11, 13–22, and 24. Sessions 2, 12, and 23 are conducted in the Laboratory of Motion Analysis at the Örebro University Hospital which is equipped with the EEG-fNIRS and Motion Capture devices. The same locations were used for the pilot study.

### 2.13. Frequency and duration

The duration of the biweekly movement intervention will be 60 min, the order and content of which is described in detail in Table 1. Throughout the intervention period, the participants will be encouraged to practice their favorite movement or relaxation techniques at home between the sessions if they want to. Each of the three parts of the intervention will be recorded, and the research team will distribute video-links upon request to the participants if they want to have support in practicing on their own at home. Moreover, when a participant fails to attend a session, an email with the video-link will be sent out, with encouragement to practice at home and keep up the frequency of two sessions a week.

### 2.14. Tailoring and modification

After the pilot study, which was conducted in spring 2023, the intervention is modified if needed, to suit the needs of the participants based on the remarks made during the pilot study. To address individual differences and to give easy-access modifications when needed, the components will be introduced in steps with sensitivity to the group needs with a central aim to make the participants feel included regardless of previous experience. For those who need extra instruction and support, one of the instructors will be able to assist.

### 2.15. Adherence

Intervention adherence will be noted by the instructors during both pilot study and main study. To stimulate high adherence to the intervention throughout all the intervention time period, we will work in close collaboration with the clinic, both for reminding about the importance of intervention participation during the 12 weeks and in practical issues regarding getting to the studio in time. To keep the creative physical exercise sessions motivating and interesting, the focus will be on enjoyment, social inclusion and increasing body movement in harmony with music. In addition, the fact that the intervention consists of three different parts with separate themes will hopefully stimulate adherence motivation. To retain as many participants as possible, reminder text-messages and emails will be sent to participants. Notes about participants who discontinue or deviate from the study will be made.

Randomization will minimize bias, but we will assess any change in the treatment plan of the participants including medication, if they stop taking their medication and become ill, or if they change to a better medication and improve because of the intervention.

## 3. Outcomes

This study includes neuroscientific and physiological measures, questionnaires (for the patients in the study and their family member or other next of kin), and qualitative interviews (with both the patients in the study and their family member or other next of kin; Table 2). The questionnaire sessions are performed at the Örebro University Hospital, and the project team provides assistance. The measures include PANSS, CGI-s, CGI-I and EQ-5D-L, PSP, and CDSS are needed to measure change in symptom presentation (PANSS), disease severity (CGI-S) and changes in that improvement (CGI-I; Busner and Targum, 2007), improvement in social functioning (PSP), quality of life (EQ-5D-L). The explorative results cover the subjective experience of the patients and their related parties regarding the participation in the intervention (qualitative interviews). See Table 2 for the timeline of the data collection of all outcome measures.

**TABLE 2:** Enrollment, interventions, and assessments.

Testing occasion	Baseline, 2–4 weeks before intervention approx. 150 min	Week 2 (session 2) approx. 60 min	Week 6 (session 12) approx. 60 min	Sometime week 5–7 approx. 60 min	Week 12 (session 23) approx. 60 min	Week 12 approx. 150 min	Month 6 and 12 (longtime follow up) approx. 150 min
**Primary outcome measure**							
PANSS negative symptoms subscale	V, O two items			V, O two items		V, O two items	V, O two items
**Secondary outcome measures**							
General PANSS and positive symptoms subscale (45 min)	V, O two items			V, O two items		V, O two items	V, O two items
CGI-S and CGI-I (5 min)	V			V, O for CGI-I		V, O for CGI-I	V, O for CGI-I
CDSS (10 min)	V			V		V	V
PSP (10 min)	V			V		V	V
Burden of care, VAS (5 min)	O			O		O	O
BAQ-sv, 10 min	X			X		X	X
EQ-5D-5L, 5 min	X			X		X	X
Weight, length, BMI, 5 min	X			X		X	X
Waistline, 1 min	X			X		X	X
Blood pressure, 10 min rest, 2 min test	X			X		X	X
Resting pulse, 1 min	X			X		X	X
Breathing frequency, 1 min	X			X		X	X
Postural stability/balance, 10 min	X			X		X	X
Chair stand test, 3 min	X			X		X	X
fNIRS-EEG combined with eye tracking during cognitive paradigms including STROOP, verbal fluency, AX-continuous performance test, Emotion regulation test. 45 min	X					X	X
Everyday activity/accelerometer	X (measured at home, 7 days)					X (measured at home, 7 days)	
Motion Capture 60 min		X	X		X		
**Exploratory outcome measures**							
Interview, 60 min						X, O	

An “X” represents a patient report, an “O” represents a family member or other next of kin report, and “V” represents a clinician report.

### 3.1. Primary outcome measure

#### 3.1.1. Positive and negative schizophrenia symptom scale (PANSS) negative symptoms subscale

The healthcare professional evaluates the patients with PANSS Negative Symptoms subscale (Cronbach’s alpha of 0.83, Kay et al., 1988; von Knorring and Lindström, 1992) at baseline, at week 6 at and after week 12, PANSS Negative Symptoms subscale will also be collected in the follow-up session; 6 and 12 months after finishing the intervention.

### 3.2. Secondary outcome measures

#### 3.2.1. Positive and negative schizophrenia symptom scale (PANSS), clinical global impression severity (CGI-S), and improvement (CGI-I)

The healthcare professional evaluates the patients with general PANSS and Positive Symptoms subscale (Cronbach’s alpha of 0.73 on the positive subscale and 0.87 for general psychopathology, von Knorring and Lindström, 1992) at baseline, at week 6 at and after week 12, Clinical Global Impression Severity (CGI-S) before session 1, at week 6 and after week 12 and Clinical Global Impression Improvement (CGI-I) at session 12 on week 6 and after week 12 are done by psychiatrist, family member or other next of kin and the patient on the same timeline. General PANSS and Positive Symptoms subscale, CGI-S and CGI-I will also be collected in the follow-up session; 6 and 12 months after finishing the intervention.

#### 3.2.2. Quality of life, social performance, and depressive symptoms

Patients will fill in quality-of-life questionnaires (EQ-5D-5L) before, after the intervention and at follow ups. The patients’ psychiatrist and a person who knows the patients, such as a family member, will evaluate the state of the patient before and after the intervention, and then, the family member will also be asked about their experience on burden of care. The Personal Social Performance scale (PSP, internal consistency reliability for the four items exceeding 0.70 for people with schizophrenia, Kawata and Revicki, 2008) assesses socially useful activities, personal and social relationships, self-care and disturbing and aggressive behaviors. The Calgary Depression Scale for Schizophrenia (CDSS, high inter-rater reliability with an intraclass correlation of 0.895 and Cronbach’s alpha 0.79 for people with schizophrenia, Addington et al., 1993) is a nine-item clinician rated outcome measure that assesses the level of depression in people with schizophrenia.

#### 3.2.3. Brain imaging

To investigate whether the InMotion intervention will enhance cognitive processes and their underlying neural functions, behavioral tests with brain imaging on attention and emotion regulation will be conducted (Balconi et al., 2015; Popov et al., 2019). Cortical activity will be measured with a combined EEG-fNIRS system (fNIRS: Sport 2, Nirx Medical Technologies, Los Angeles, CA, USA; EEG: G.tec medical engineering GmbH, Schiedlberg, Austria). The cap will have 16 fNIRS sources and 16 detectors over the frontal, and parietal brain regions to measure cognitive and motor activity, respectively. The cap will also have 64 EEG electrodes distributed throughout the head (10–20 system; Jasper, 1958). Eye tracking glasses have small cameras to detect the movements of the pupils, amount of saccades and dilation of the pupil. Eye-tracking (Levy and Gooding, 2010; Morita et al., 2017, 2019; Wolf et al., 2021) and EEG-fNIRS will be used to evaluate cognitive functioning.

Patients will conduct the following tasks during brain imaging and eye tracking: (a) cognitive tests with region of interest (ROI) on the prefrontal cortex: Stroop task (cognitive interference; Stroop, 1935; MacLeod, 1991), expectancy AX Continuous Performance Test (AXCPT; context processing; Ochsner et al., 2002; Barch et al., 2003; Braver, 2012), verbal fluency (cognitive paradigms related to prediction error as described above), and (b) emotion regulation with ROI on the frontal cortex: induction and regulation with specific positive (happiness) and negative (disgust) emotions.

#### 3.2.4. Motion capture

Movement of the participants will be recorded with the Qualisys Motion Capture system consisting of 12 cameras (Arqus A12, Qualisys AB, Gothenburg, Sweden) during the movement intervention on sessions 2 on week 1, 12 on week 6 and 23 on week 12. Each participant and the facilitators will have reflective markers attached to the skin on their hands, knees and elbows (six sensors in total per person) which they will wear throughout the movement session. The markers are small and light and will not influence movement.

#### 3.2.5. Body awareness

Patients will also fill in Body Awareness Questionnaire (BAQ; Shields et al., 1989) before, after the intervention and at follow ups.

#### 3.2.6. Physical measures

Physical function and status will be measured at baseline, half-time through the intervention, after the intervention and at follow up 6 and 12 months. The measures are the following: (1) Postural stability test (Van der Heijden et al., 2019) with participant standing on a force plate, (2) chair stand test (Jones et al., 1999) to measure leg strength, (3) weight, length, BMI and waistline, (4) blood pressure measured by blood pressure monitor, (5) resting heart rate, (6) breathing frequency, and (7) everyday activity which is measured by an accelerometer which the participants will wear around their waist for 7 days in a row to collect the amount of time spent sedentary vs. active, and the intensity of the activity.

#### 3.2.7. Qualitative interviews

To capture the participants’ experiences of taking part of the intervention, individual semi structured interviews on the topic will be conducted with 15–20 participants in the intervention groups (Bryl et al., 2020). The sample size has been set with the intention to catch a width of different experiences. The interviews will be conducted immediately after the last session, to have the experience as fresh as possible. The participants self-selected expressions, own reflections and insights will add to the quantitative measures and contribute to a wider understanding of the phenomenon. The questions concern how the participants experienced being part of the InMotion intervention, whether and how the intervention influenced their health and everyday life, as well as reflections on different perspectives such as the group format. Moreover, the family member or other next of kin of these participants will also be asked to participate in an interview. The interviews will be face to face, conducted by the research team, and get to share their experience of the intervention as well as eventual effects from it.

### 3.3. Data analysis

#### 3.3.1. EEG-fNIRS analysis

EEG data will be analyzed with EEGLAB (Delorme and Makeig, 2004) for MATLAB for amplitude power and phase synchrony (Tass et al., 1998). fNIRS data will be analyzed with Satori.^1^

#### 3.3.2. Motion capture analysis

Motion Capture data will be analyzed with the MoCap toolbox (Burger and Toiviainen, 2013) which is a set of functions designed for MATLAB. The movement quantity, to measure changes in mobility, and movement quality on the smooth-jerk axis, to measure changes in rigidity of movement, will be computed (Burger and Toiviainen, 2013; Poikonen et al., 2018; Bryl and Biondo, 2021).

#### 3.3.3. Statistical analysis

The InMotion study follows the intention-to-treat paradigm. Complementary analyses will be performed per protocol according to the number of sessions the participant attends. For baseline statistics between the groups, descriptions will be constructed using frequencies and proportions for categorical data and means and standard deviations for continuous variables. Baseline characteristics will be compared using the Fisher exact test for dichotomous outcomes, the Mantel-Haenszel chi-square test for ordered categorical outcomes, and the Mann-Whitney *U*-test or unpaired *t*-test for continuous outcomes. A *P*-value of < 0.05 for the two-tailed test will be considered statistically significant for all outcomes. When deemed necessary, correction for multiple significance will be performed.

The primary outcome analysis will initially be performed using a Fisher exact test to evaluate differences between groups. To analyze the change in scores from baseline between groups for approximately normally distributed variables, a repeated-measures covariance pattern mixed model will be used, adjusting for significant differences at baseline. To identify predictive factors at baseline that might be associated with the primary outcome, a univariable logistic regression analysis followed by a stepwise multiple logistic regression analysis will be performed.

The distribution of continuous variables will be described using the mean and standard deviation or median and interquartile range. Categorical variables will be described with numbers and percentages.

The interviews will be analyzed by qualitative content analysis, to this day planned to be according to Hsieh and Shannon (2005); data from interviews will be transcribed verbatim, read through and analyzed into units. These units will be condensed and coded. The codes will be divided into categories and subcategories, and abstracted to overall themes if found. Latent meaning will be interpreted and the themes shall capture the core message of the whole data-material and will be strengthened with quotes. NVivo software program (QSR International; NVivo, 2022) will be used during the analysis.

## 4. Ethics and dissemination

The study is conducted in accordance with the standards of Good Clinical Practice and in agreement with the Declaration of Helsinki. Written informed consent will be provided by the research team. The information letter includes written information about the study, the purpose and procedures, the voluntary nature of participation, and the option to withdraw at any time. If needed, clinicians will help to clarify the written information. The participants are guaranteed confidentiality and that their data are stored on a secured server except the brain imaging data, which will be made anonymously public. It is impossible to identify the participants from the brain imaging data, and at the same time, open science is supported by publishing the data. The participants will be informed about the public data sharing on a group level without any personal information in their information letter and asked for their consent for it. In addition, the participants in the study were invited to an information meeting before the start of the study, where verbal explanations of all the procedures were given. Any adverse events or harm arising from study participation will be reported and managed by the instructors and the research team in accordance with ordinary healthcare routines.

Data are collected in both pen-and-paper and digital formats and securely stored at the University Health Care Research Center. The questionnaires and tests will be pretested on a conditions-appropriate group during the pilot study to determine whether the wording and length of the questionnaire and tests were appropriate for the study’s target group. The pilot study investigated different aspects of intervention fidelity. No unauthorized persons have access to the collected data, during the study, apart from the brain imaging data as mentioned above. All the data collected are coded to protect the study participants’ identification. All data and the code list are stored securely and separately to prevent unauthorized persons from having access to them.

## 5. Adverse event and safety monitoring

Instructors in the study will regularly ask participants in the intervention how they felt after the previous session. Any adverse events related to the participation in intervention will be recorded and assessed in terms of severity which will be reported to the principal investigator, serious adverse events will be recorded in the patient’s medical chart.

## 6. Results

The pilot study, which was conducted in spring 2023, evaluated the acceptability and feasibility of different perspectives of the intervention, the data collection timeline, and the logistics of the study. This gives the research team valuable lessons to learn and if needed, enable adjustments before entering the main study. Due to the small sample size in the pilot study, we do not evaluate the feasibility in terms of recruitment, measurement completion, attendance rate nor drop-out. We will include the adjustments in the intervention design, data collection and logistics which will be made based on the pilot study and advance to the main RCT study in autumn 2023. We expect to publish the first results of the pilot study in spring 2024.

We expect to publish the first results of the main study in 2026 with the primary, secondary and exploratory results described in detail in section “3. Outcomes.”

## 7. Discussion

Schizophrenia is prevalent in the adult population worldwide. Considering that the pharmaceutical treatment of the illness is far from optimal, cost-effective and easy-access interventions, which can be added on the conventional treatment, are needed. Physical, creative and social activities form an effective strategy, but intervention studies are needed to better understand if and how such activities improve the clinical symptoms, quality of life, physical skills, cognitive and emotional capacities, and facilitate regular participation for this target group.

Our study will investigate several aspects of the ways that mixed exercise program with creative movement may improve symptoms, physical health and quality of life of adults with schizophrenia or schizophrenia-like symptoms. As primary results, we expect to find differences related to the negative symptoms of schizophrenia. As secondary results, we expect to find differences in several variables on general and positive symptoms, physical health, cognitive and emotional capacity and quality of life. To our knowledge, this is the first study to investigate the influence of regular physical exercise with focus on creative, emotional and social activity in people with schizophrenia with a broad spectrum of measures from clinical symptoms to physical health and brain imaging.

Several studies report improved physical and emotional state in people with schizophrenia after regular practice of physical and body-awareness training, respectively (Martin et al., 2016, 2017; Girdler et al., 2019; Millman et al., 2021). However, neuroscientific results for such interventions are still lacking. We aim to connect the clinical and physical outcomes to the outcomes of brain imaging.

The challenge in a holistic intervention combining physical, creative and social components is that we cannot know which individual components contribute to the changed clinical and health outcomes. The influential subcomponents in a positive outcome could be the supportive group setting, instructors, music, or specific parts of movement exercises. However, we hypothesize that the core of the intervention is the *combination* of all these subcomponents together. Methodologically, we follow the highest standards for clinical studies including the randomized controlled design, one-year follow-up and the combination of quantitative and qualitative measures related to clinical symptoms, physical abilities, and cognitive, emotional and cerebral functions.

Several aspects of our trial design are worth noting as potential limitations. Since the sample size is calculated according to the primary outcome PANSS, the results of the secondary outcomes should be considered cautiously, and the results replicated in future studies. If the sample size was calculated according to the amount of all the measurements, the sample size would be so high that it would be beyond realistic possibilities, and this study would very likely never be completed. Some of the measures, such as PANSS, are subjective whereas other measures, such as brain imaging, are objective. Although it is difficult to blind the assessor during the assessment of the objective measures, the person performing the analysis will be blinded. Since this is the first time we use such intervention of creative physical exercise, we cannot know whether the design will be robust enough to capture changes with the content (e.g., physical activity increasing heartrate, social interaction, body awareness) and dosage we defined (twice a week for 60 min per session). However, the content and dosage are based on previous literature on creative movement interventions in schizophrenia (Martin et al., 2016; Cheng et al., 2017; Bryl et al., 2020).

In the pilot study, we tested the feasibility of biweekly attendance, which might be challenging for the participants with schizophrenia. However, in the pilot study, there was a high attendance rate and no dropouts. Nonetheless, we are aware that biweekly commitment implies a behavioral change that can be hard to achieve for this target group. Thus, it is interesting to investigate if the format of this intervention can influence motivation, retention, and interest to participate regularly.

The results from this study will broaden the knowledge of the ways non-pharmacological interventions can be valuable for reducing the burden of schizophrenia for patients themselves, their families, and the healthcare system. Mixed physical exercise with creative movement could be an example of an easy-access joyful intervention as a complementary treatment.

To conclude, this randomized controlled study with 60 participants will investigate the effects of combined physical, creative and social training in adults with schizophrenia. The primary aim is to study the effects of the intervention on negative symptoms of schizophrenia, but several other aspects, such as physical skills and health-status, cognitive and emotional capacity and brain functions related to them as well as movement quantity and mimicry, will also be studied. The results from this intervention study may provide useful information and potentially a complementary treatment to use for caregivers in primary healthcare and psychiatric in- and outpatient clinics.

## Ethics statement

The studies involving human participants were reviewed and approved by the Swedish Ethical Review Board in Uppsala, Sweden (No. 2022-03980). The patients/participants provided their written informed consent to participate in this study.

## Author contributions

HP, AD, ML, and MMs designed the creative movement intervention. HP wrote the original manuscript. All authors commented and edited the manuscript.

## Footnotes

1
www.nirx.net/satori
